# Motion correction in MR image for analysis of VSRAD using generative adversarial network

**DOI:** 10.1371/journal.pone.0274576

**Published:** 2022-09-14

**Authors:** Nobukiyo Yoshida, Hajime Kageyama, Hiroyuki Akai, Koichiro Yasaka, Haruto Sugawara, Yukinori Okada, Akira Kunimatsu

**Affiliations:** 1 Department of Radiology, Institute of Medical Science, The University of Tokyo, Minato-ku, Tokyo, Japan; 2 Division of Health Science, Graduate School of Health Science, Suzuka University of Medical Science, Suzuka-city, Mie, Japan; 3 Department of Radiology, The University of Tokyo Hospital, Bunkyo-ku, Tokyo, Japan; 4 Department of Radiology, Tokyo Medical University, Shinjuku-ku, Tokyo, Japan; 5 Department of Radiology, Mita Hospital, International University of Health and Welfare, Minato-ku, Tokyo, Japan; Harvard Medical School, UNITED STATES

## Abstract

Voxel-based specific region analysis systems for Alzheimer’s disease (VSRAD) are clinically used to measure the atrophied hippocampus captured by magnetic resonance imaging (MRI). However, motion artifacts during acquisition of images may distort the results of the analysis. This study aims to evaluate the usefulness of the Pix2Pix network in motion correction for the input image of VSRAD analysis. Seventy-three patients examined with MRI were distinguished into the training group (n = 51) and the test group (n = 22). To create artifact images, the k-space images were manipulated. Supervised deep learning was employed to obtain a Pix2Pix that generates motion-corrected images, with artifact images as the input data and original images as the reference data. The results of the VSRAD analysis (severity of voxel of interest (VOI) atrophy, the extent of gray matter (GM) atrophy, and extent of VOI atrophy) were recorded for artifact images and motion-corrected images, and were then compared with the original images. For comparison, the image quality of Pix2Pix generated motion-corrected image was also compared with that of U-Net. The Bland-Altman analysis showed that the mean of the limits of agreement was smaller for the motion-corrected images compared to the artifact images, suggesting successful motion correction by the Pix2Pix. The Spearman’s rank correlation coefficients between original and motion-corrected images were almost perfect for all results (severity of VOI atrophy: 0.87–0.99, extent of GM atrophy: 0.88–00.98, extent of VOI atrophy: 0.90–1.00). Pix2Pix generated motion-corrected images that showed generally improved quantitative and qualitative image qualities compared with the U-net generated motion-corrected images. Our findings suggest that motion correction using Pix2Pix is a useful method for VSRAD analysis.

## Introduction

Alzheimer’s disease (AD) is the most common form of dementia [1], and hippocampal atrophy from neurodegeneration is an important morphological change that is useful in diagnosing AD in clinical practice [2]. Atrophy assessment using magnetic resonance imaging (MRI) is considered a valid method for the evaluation of AD status and progression [3]. Voxel-based morphometry (VBM) is a method that transforms and spatially normalizes MRI image data based on standard brain coordinates to perform whole-brain morphological analysis automatically [4]. A voxel-based specific region analysis system for Alzheimer’s disease (VSRAD), which is a software that measures the atrophy of the hippocampus by setting a region of interest for AD in VBM, is widely used for the diagnosis of AD in clinical practice [5]. VSRAD can evaluate the atrophy of the hippocampus and para-hippocampus but also the reduction in the regional volume by comparing brain MRI images from patients and healthy subjects in a database, and its diagnostic accuracy for detecting very mild AD has been reported to be as high as 91.6% [6]. The acquired image data used for the VSRAD analysis require the entire head to be included in the imaging area with a thin slice thickness of 1.0 to 1.5 mm with no gap, using three-dimension T1-weighted images (3DT1WI) [2]. However, the acquisition time of 3DT1WI is long and may result in artifacts due to spontaneous body movements. These motion artifacts affect the size of brain structures, and thus pose a problem for VSRAD analysis [7].

The following three methods have been proposed to correct motion artifacts in MRI images. The first method is to set up a motion correction, such as periodically rotated overlapping parallel lines with enhanced reconstruction, in the MRI imaging sequence [8]. The second method is to attach an external device, such as an optical camera, to the patient and estimate the motion [9]. The third method is to use convolutional neural networks (CNN) or generative adversarial networks (GAN) to correct the obtained MRI image [10, 11]. Compared with the other motion compensation methods, CNN or GAN are perhaps more simple methods because they do not require any changes in the acquisition sequence or image reconstruction method, nor do they require any external hardware devices. Motion correction using CNN and GAN has been investigated in brain MRI but also in cardiac [12] and abdominal MRI [13], which are also affected by respiration and heartbeat. CNN learns to minimize the loss function, and while the learning process is automatic, a lot of manual work is required to design an effective loss. Meanwhile, GAN learns a loss function that tries to classify the output image as either real or fake, and simultaneously learns a generative model that minimizes this loss [14]. One type of such a GAN is Pix2Pix, a network dedicated to image-to-image conversion [15] that has recently been evaluated using clinical images [16]. Pix2Pix is a network consisting of U-Net as a generator and PatchGAN as a discriminator.

To the best of our knowledge, there have been few studies using GAN and CNN motion-correction techniques for VBM [17]. In particular, no studies have used these techniques for VSRAD analysis. We hypothesized that the motion-corrected images using Pix2Pix could accurately measure hippocampal atrophy for VSRAD analysis. Thus, the purpose of the present study was to evaluate the usefulness of the Pix2Pix network in motion correction for the input image of VSRAD analysis.

## Materials and methods

### Participants and image acquisition

The present study retrospectively analyzed 78 consecutive Japanese patients who visited the research hospital, Institute of Medical Science, the University of Tokyo from April 2013 to April 2020 and underwent MRI scans for suspected dementia. The Research Ethics Committee of the Institute of Medical Science, University of Tokyo, approved the study protocols (2019-41-1031), and the requirement for written informed consent was waived.

**Fig 1:**
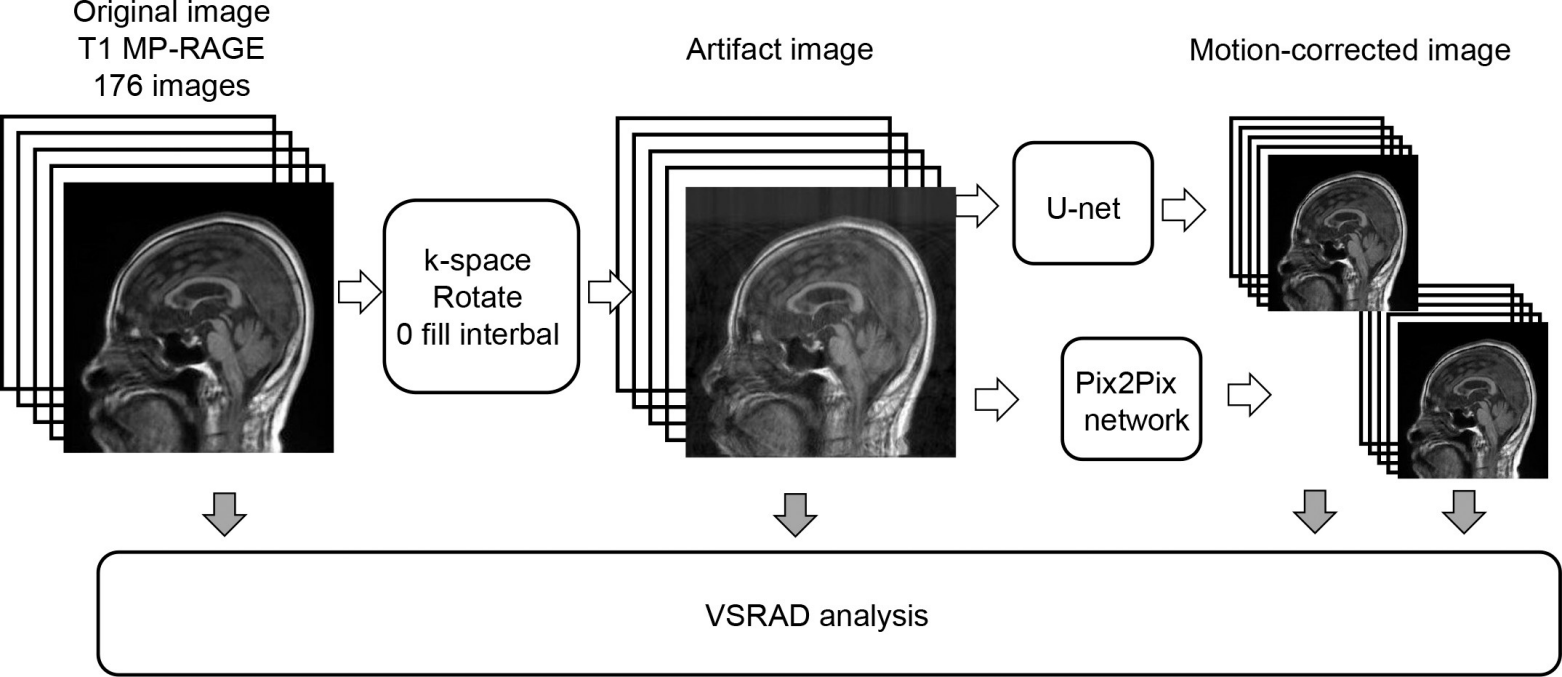
Schematic diagram of this study. Artifact images were created by manipulating the k-space of the original images. Motion artifact suppressed images were generated from the artifact images using the Pix2Pix network. VSRAD analysis was performed on three types of images: the original image, the artifact image, and the motion-corrected image. Then, differences among images of the VSRAD analysis results were examined.

Whole brain images were acquired using a 3.0 Tesla clinical MRI scanner (MAGNETOM, Skyra, Siemens AG, Healthcare Sector, Erlangen, Germany) with Syngo MR E11 software and a 20-channel head matrix coil. The VSRAD images were acquired using a T1-weighted, magnetization-prepared rapid gradient-echo sequence with 176 sagittal slices (echo time = 2.04–2.09 ms, repetition time = 1.8 s, flip angle = 10 degrees, inversion time = 800 ms, slice thickness 1.0 mm, no gap, matrix 256×256, field of view 230–240 mm, number of excitations = 1, acquisition time = 4 min 43 s). Following review by two experienced radiologists, five patients were excluded due to motion artifacts in their images, and the remaining 73 patients were divided into training (n = 51, April 2013 to December 2017) and test (n = 22, January 2018 to April 2020) groups. Ten percent of the training group was assigned to the validation group. The workflow of this study is illustrated in Fig 1.

T1-MPRAGE (T1-weighted, magnetization-prepared rapid gradient-echo sequence); VSRAD (Voxel-based specific region analysis system for Alzheimer’s disease).

### Creation of artifact images

**Fig 2:**
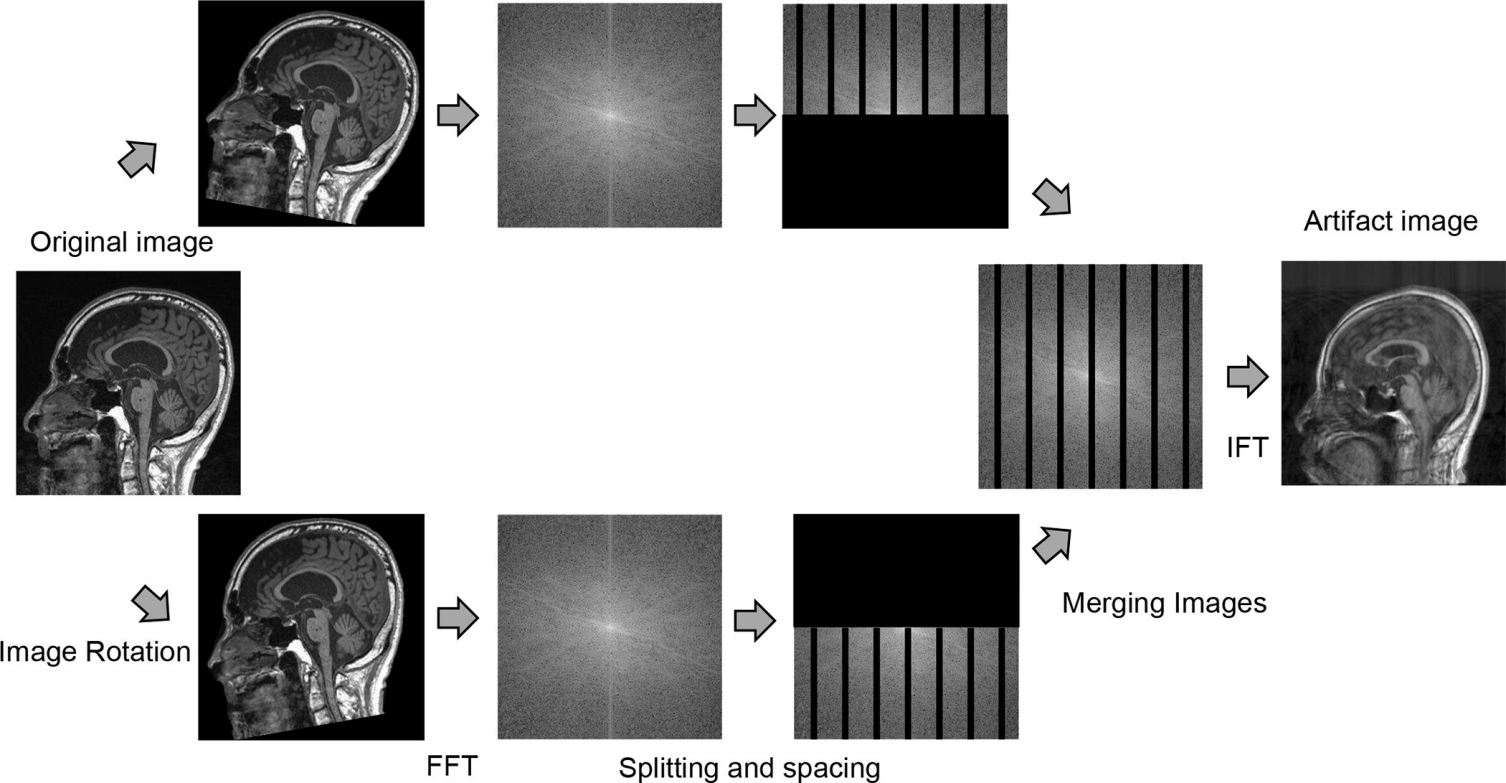
Creation of motion artifact images. The following process was used to create motion artifact images. First, two images were created, one rotated to the left and one rotated to the right of the image. Secondly, the two types of images were Fourier transformed (FFT), and we filled k-space with zeros at equal intervals. Finally, the top and bottom half of each k-space were merged to create one image, and we performed an inverse Fourier transform (IFT) of the merged image to create an image with simulated motion artifacts.

**Fig 3:**
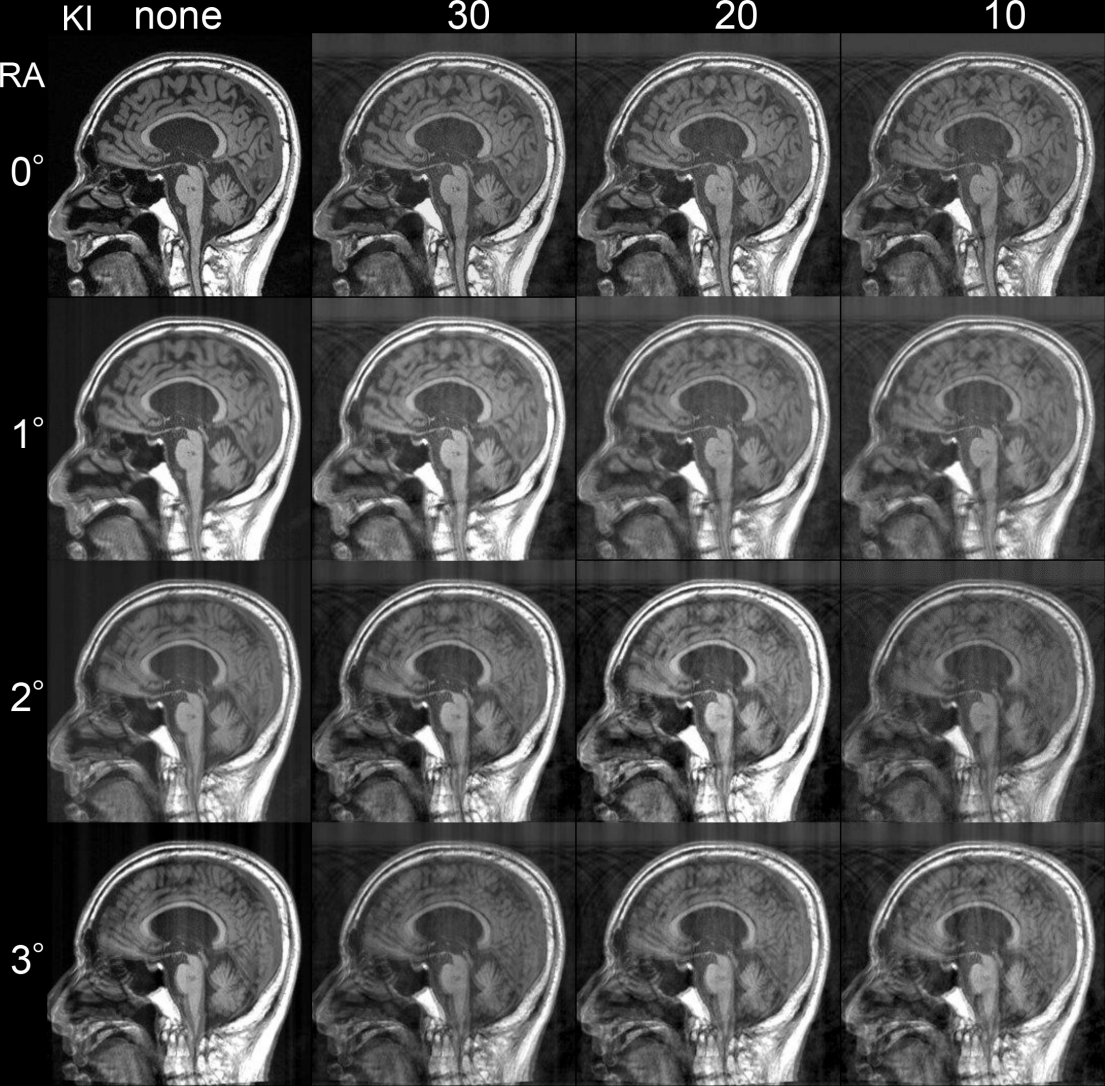
Training data sets. A total of 16 artifact images were created with four rotation angles (RA) and four patterns of k-space zero-fill intervals (KI). In the artifact images, the larger the rotation angle, or the smaller the zero-fill interval in k-space, the stronger the motion artifact. In the present study, we created a training data set by pairing each of the 16 artifact images with the original image. We demonstrate an example used as the input image for the training group.

In the present study, we used a computer with 3.6GHz/s (corei7–7700), 48GB/s memory bandwidth, and a GeForce GTX 1070 graphics card (Nvidia Corporation, Santa Clara, CA, USA) with 8GB memory per board. Images with simulated motion artifacts were modeled in reference to the study of Lucilio *et al*. [18]. The creation of motion artifacts was performed using MATLAB software R2021a (Mathworks, Natick, Massachusetts, USA). In the training group, we created sixteen patterns of artifact images (including the original image) assuming vertical rotational and anterior-posterior-motion movements in the sagittal plane of the brain. All combinations of four rotation angles, including zero and four patterns of zero-filled intervals on k-space, were created. First, we rotated the image. The rotation angle was selected from 0, 1, 2, or 3 degrees for each patient, and two images were created by rotating the same angle to the left and right. Secondly, the two types of images were Fourier transformed, filling them with zeros at equal intervals. This interval was selected from 0, 10, 20, or 30 pixels for each patient. Finally, the top and bottom half of each k-space were merged to create one image, and we performed an inverse Fourier transform of the merged image to create an image with simulated motion artifacts (artifact image) (Figs 2 and 3). For the training group, we used a total of 47872 (= 51 patients×176 slice×16 motion pattern×1/3 (adjusting number of images to meet with our computer specs)) pair image data sets created at three slice intervals of the total artifact image.

Using the same method with the training group, artifact images were generated for the test group with four fixed patterns 0_none (equal to the original image), 1°_30, 2°_20, and 3°_10 (rotation angle _ k-space interval). In addition, we created two patterns of intervals 1.5°_25, 2.5°_15 (rotation angle _ k-space interval) that were not trained with Pix2Pix.

### Pix2Pix network

We used the Pix2Pix network to generate motion artifact corrected images. We used the Python 3.6.10 with Anaconda 3.0 distribution software (Python Software Foundation, Delaware, USA), TensorFlow-GPU 2.1.0, and Keras 2.3.1 (Google, Mountain View, Calif, USA).

**Fig 4:**
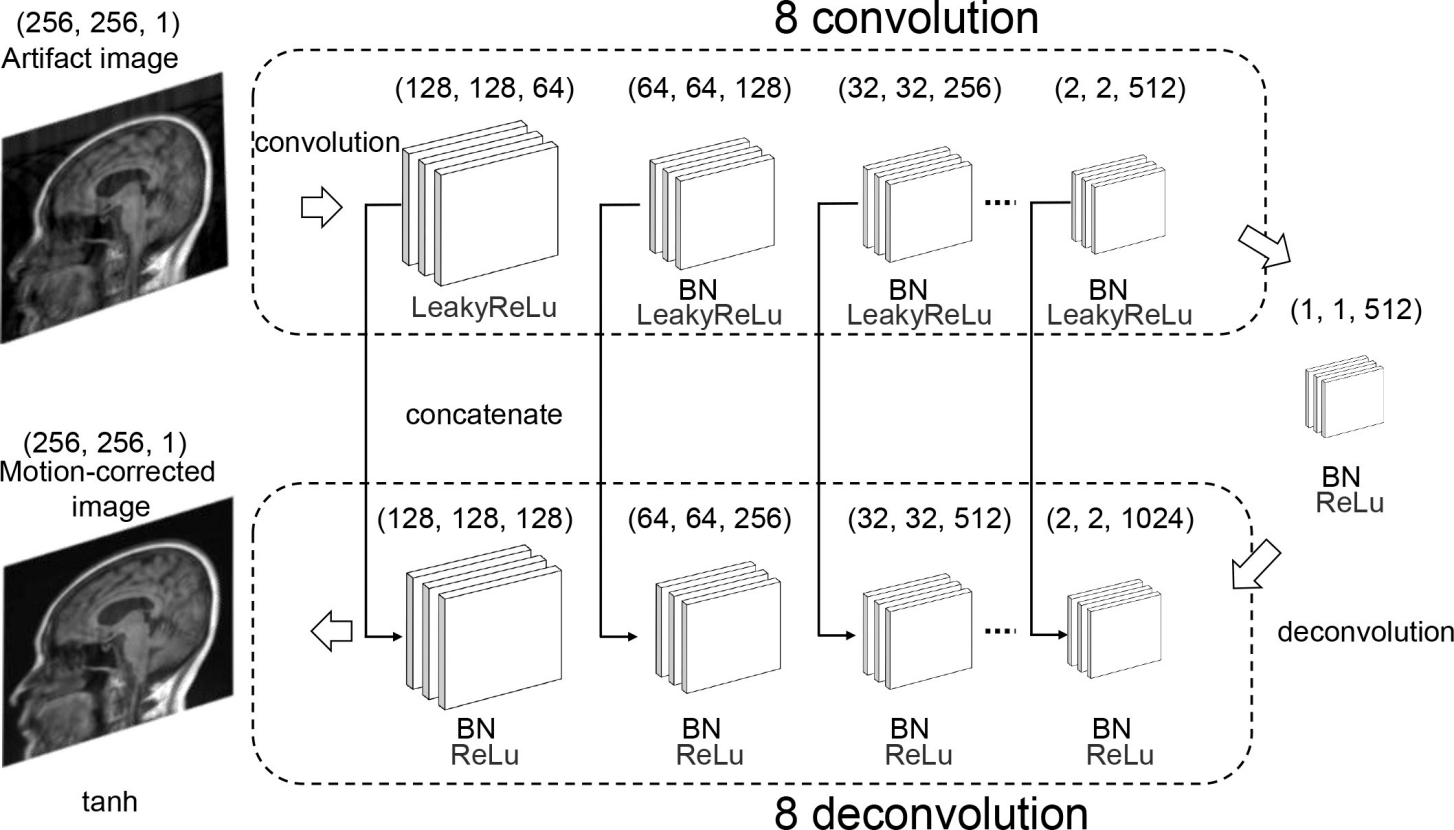
Generator network. We used a U-Net-based network for the generator network. This network comprised an encoder consisting of eight convolutional layers and a decoder consisting of the same number of deconvolutional layers. For the convolutional layer’s input and output, activation by the LeakyReLu function and batch normalization (BN) were performed in the encoder stack, whereas activation by the ReLu function and BN was performed in the decoder stack. The motion-corrected image was the output of the decoder stack activated by the tanh function.

For the Pix2Pix generator network, we used a U-Net-based network [19] with a structure that passes information between encoder-decoders, whose encoder stacks consist of eight convolutional layers and decoder stacks consist of the same number of deconvolutional layers that employed upsampling and convolution. In the encoder stacks, the LeakyReLu activation function was applied to the input of the second and subsequent convolutional layers, and batch normalization was applied to the output. Similarly, the ReLu activation function was applied to the input of the deconvolutional layer in the decoder stacks, and batch normalization was also applied to the output. The tanh activation function was applied to the output of the final convolutional layer to produce the motion-corrected image (Fig 4).

**Fig 5:**
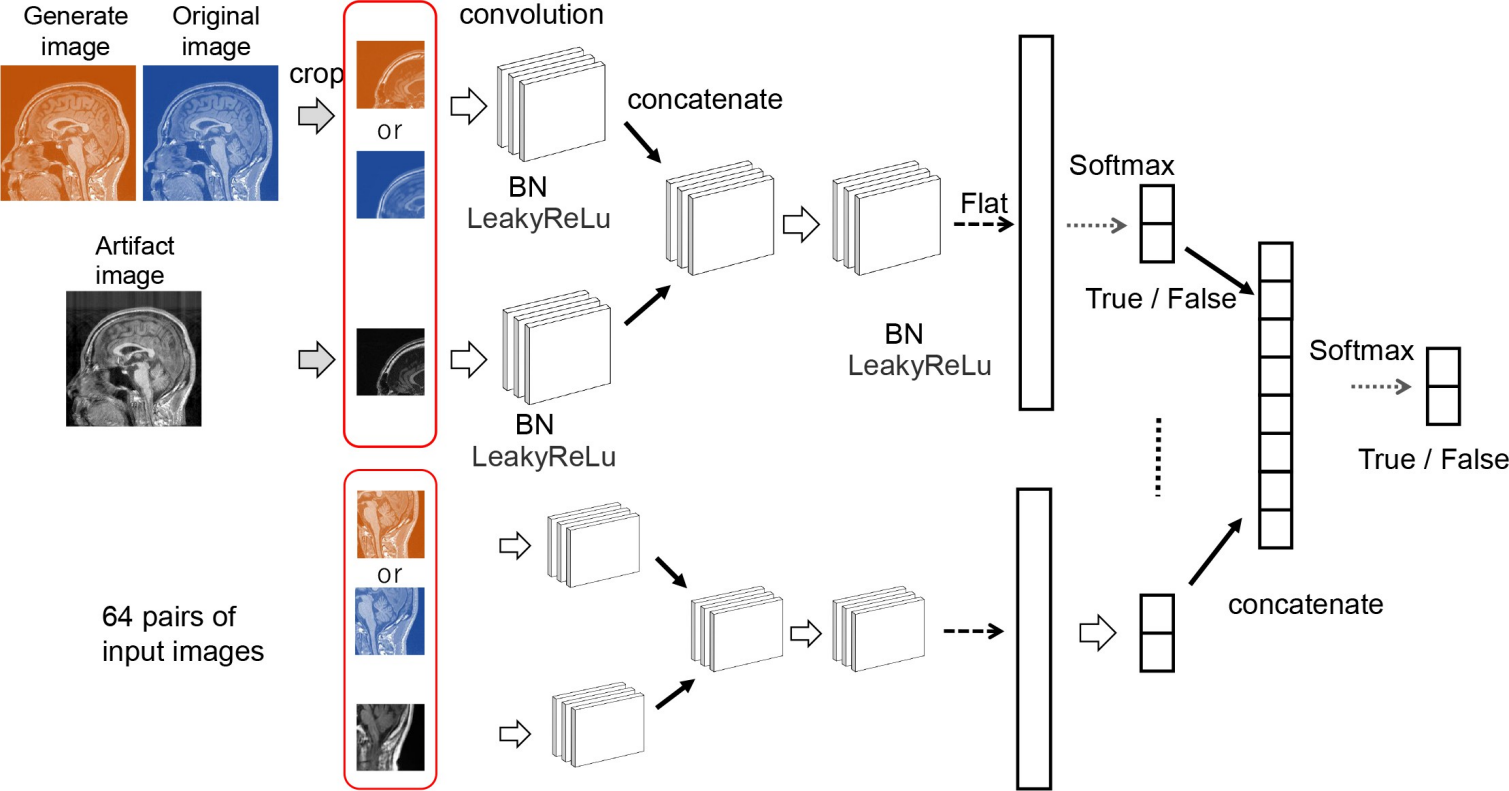
Discriminator network. PachGAN was used as the Discriminator network. First, the input image was cropped into small regions (patch size). Secondly, for each small region, binary classification was performed by a network consisting of a convolutional layer, an activation layer (LeakyReLu, Softmax), a batch normalization layer (BN), and an affine layer (Flat). Thirdly, the outputs of the binary classification in all subregions were combined. Finally, binary classification was performed using the Softmax function to evaluate whether the two input images were entirely equal.

The Pix2Pix discriminator network introduces PatchGAN that evaluates whether the original image and the motion-corrected image are equivalent in a small region. This network does not directly compare the two images. Instead, it splits each image into small regions of a specific size (patch), evaluates each pair (True pair and False pair), and finally performs binary classification. The use of PatchGAN has been reported to show high learning accuracy for high-frequency components of images [20]. In the present study, the patch size was set to 32×32. Therefore, 64 paired patch images are input in the discriminator at once (matrix size (256 x 256) / patch size (32 x 32) = 64 paired images). First, the original image and the motion-corrected image for each patch were convolved once. Second, the output for each patch was concatenated and convolved, and was then transformed to one dimension for full combination and binary (True or False) classification with the Softmax function. Finally, all patches were concatenated and classified as binary (True or False) by the Softmax function (Fig 5).

The learning parameters were trained using an Adam optimizer with a constant learning rate of 1e-4, beta_1 = 0.9, and beta_2 = 0.999 [21]. The mini-batch size was set to 32. The order of the data was randomized in each epoch, and we set the number of epochs to 100. The loss function for the Pix2Pix generator network was L1 regularization and binary cross-entropy for updating parameters, and the discriminator network used binary cross-entropy.

### U-Net

In the present study, the comparison between Pix2Pix and CNN was performed with U-Net, which was used in the generator network of Pix2Pix. The training parameters were set the same as for the Pix2Pix generation network, except that the mini-batch size was 16. The loss function for this network was set to Mean Squared Error.

### Image quality evaluation

For quantitative evaluation, we used the structured similarity indexing method (SSIM) to compare the structural and feature similarities between the original images and the artifact or motion-corrected images. Analysis of SSIM was performed using MATLAB software R2021a.

For qualitative evaluation, two diagnostic radiologists evaluated the artifact images and motion-corrected images (U-Net and Pix2Pix) generated in this study using the following 5-point Likert scale for the degree of motion artifact.

5: No influence of movement

4: Slightly affected by motion but does not affect analysis

3: Lower limit of acceptable motion effect

2: Motion influence is present and may affect the analysis.

1: Cannot be analyzed due to the effect of motion.

The radiologists were blinded to the image acquisition method and independently assessed the subjective image quality. The data were analyzed randomly to reduce recall bias.

### VSRAD analysis

Multiplanar reconstruction was performed on the original image, artifact image, and the motion-corrected image in the 1-mm coronal plane. These three types of images were analyzed using the VSRAD advance software package (version 6.00, Eisai, Tokyo, Japan). Three factors were analyzed:

First, the severity of VOI atrophy was the Z-score of the severity of gray matter (GM) atrophy in the region of interest in AD, and it was calculated using the following equation:

(severity of VOI atrophy) = ((normal control average of voxel-level—patient’s voxel-level)/normal controls standard deviation (SD)).

Second, the extent of GM atrophy referred to the extent of GM atrophy in the whole brain (extent of GM atrophy) = a percentage of voxels with a Z-score>2 compared with the whole brain.

Third, the extent of VOI atrophy reflected the extent of GM atrophy in the VOI of AD, and it was calculated as follows:

(extent of VOI atrophy) = ((number of voxels judged to have a Z-score of more than 2/number of all voxels in the volume of the hippocampus)×100%) [6, 22].

### Statistical analysis

The male-to-female ratio was compared between the training and the test groups using the Fisher test. Age, severity of VOI atrophy, extent of GM atrophy, and extent of VOI atrophy were compared between the training and the test groups using the Mann-Whitney *U* test. The results of SSIM analysis were compared using paired *t*-tests, and qualitative image quality was compared using Wilcoxon signed-rank test. A weighted *kappa* test was used to examine the inter-observer agreements of qualitative image quality evaluation. A *kappa* value of 0.20 was defined as a slight agreement, 0.21–0.40 as fair agreement, 0.41–0.60 as moderate agreement, 0.61–0.80 as strong agreement, and 0.81–1.00 as almost perfect agreement. In the Bland-Altman analysis, the mean bias of the VSRAD analysis in the artifact *vs*. the original image, U-Net motion-corrected *vs*. the original image, and the Pix2Pix motion-corrected *vs*. the original image were plotted against the difference of the values, and the limit of agreement between the two measurements was defined as the mean±1.96 times SD of the difference with a 95% confidence interval. The results of VSRAD analysis were tested for severity of VOI atrophy, extent of GM atrophy, and extent of VOI atrophy. Four trained patterns (0°_none,1°_30, 2°_20, 3°_10) and two non-trained patterns (1.5°_25, 2.5°_15) were tested. In addition, Spearman’s rank correlation coefficients were calculated for the VSRAD measurements of the artifact *vs*. the original images, U-Net motion-corrected images *vs*. original images, and the Pix2Pix motion-corrected *vs*. original images.

These statistical analyses were performed using the EZR software [23]. The significance level was set to 0.05.

## Results

The number and mean±SD age of the patients in the training group was 51 (male: female, 20:31) and 75.24±12.16 years old, respectively. There were 22 patients in the test group (male:female, 6:16), and the mean age was 75.00±11.88 years old. The mean values of severity of VOI atrophy, extent of GM atrophy, and extent of VOI atrophy were 1.23±0.87, 5.62±3.21, and 14.83±21.08 in the training group and 1.48±1.17, 5.77±2.99, and 25.96±32.56 in the test group. There were no significant differences in sex, age, severity of VOI atrophy, extent of GM atrophy, and extent of VOI atrophy between the training and the test groups (Table 1).

**Table 1:** Details of the training and test groups.

	Training	Test	*p*-value
		(Original image)	
**Number of Patients**	51	22	
**Sex (M: F)**	20:31	6:16	0.43
**Age (years) (mean±SD)**	75.24 ± 12.16	75.00 ± 11.88	0.94
**Severity of VOI atrophy**	1.23 ± 0.87	1.48 ± 1.17	0.79
**(mean±SD)**			
**Extent of GM atrophy**	5.62 ± 3.21	5.77 ± 2.99	0.86
**(mean±SD)**			
**Extent of VOI atrophy**	14.83 ± 21.08	25.96 ± 32.56	0.48
**(mean±SD)**			

No significant differences in gender, age, and severity of VOI atrophy were observed. Standard deviation (SD); Voxel of interest (VOI); gray matter (GM).

**Fig 6:**
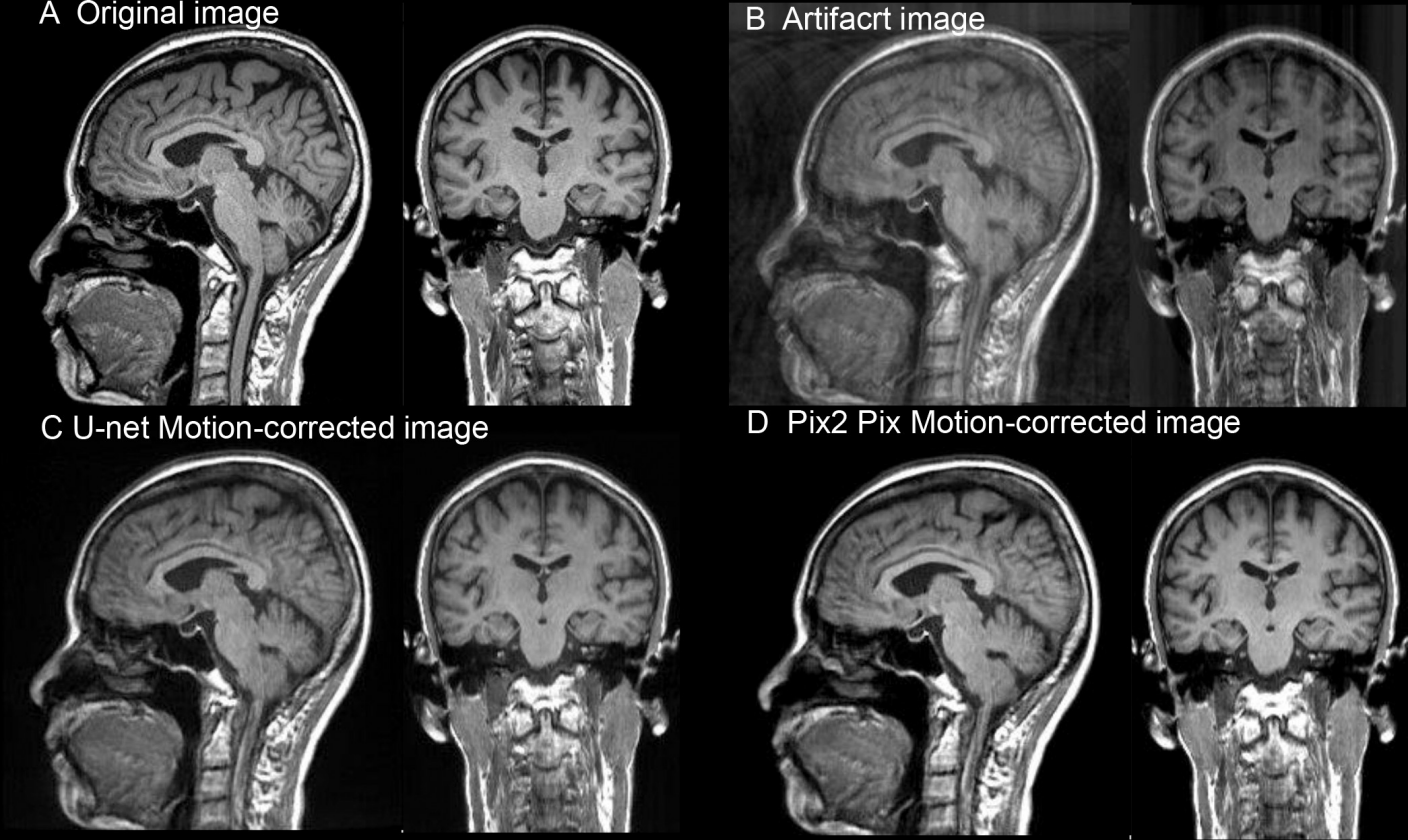
Examples of the original image, artifact image, and motion-corrected image. (A) The original T1-weighted, magnetization-prepared rapid gradient-echo sequence (T1-MPRAGE) images, (B) artifact images created by manipulating the k-space of T1-MPRAGE, (C) motion-corrected images by the U-Net network from the artifact images and (D) motion-corrected images by the Pix2Pix network from the artifact images, are shown, respectively. The rotation angle and k-space interval of the artifact images were 2°_20 pixels.

An example of the original image, artifact image, and motion-corrected image is shown in Fig 6.

**Fig 7:**
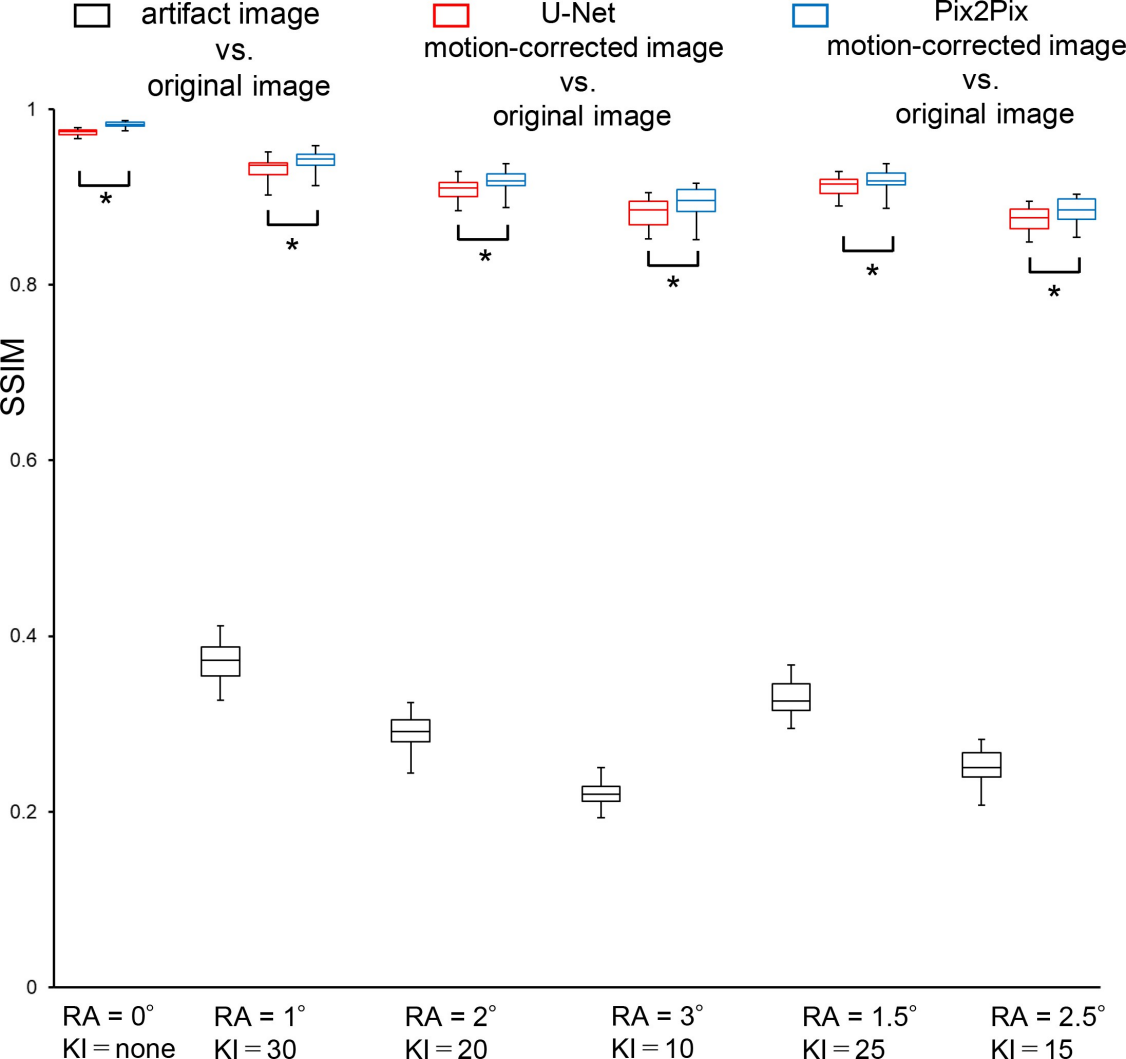
Comparison of SSIMs in the original and artifact images or generated images by the U-net or Pix2Pix. The measured SSIM values are shown in a box-and-whisker diagram. A comparison of the artifact image *vs*. the original images are shown in black, the motion-corrected image created by U-Net *vs*. the original images are shown in red, andPix2Pix-corrected *vs*. the original images are shown in blue. The SSIMs of the motion-corrected images were compared by paired *t*-test with the same rotation angle and k-space interval, and Pix2Pix was significantly higher than U-Net (*p* < 0.05).

The results of the SSIM analysis for artifact images, U-Net motion-corrected images, and the Pix2Pix motion-corrected images against the original image are shown in Table 2 and Fig 7. The mean SSIM values of the artifact, U-Net, and Pix2Pix images against the original images were 0.22–0.37, 0.88–0.97, and 0.89–0.98, respectively. We used a paired *t*-test with the same rotation angle and k-space interval to evaluate the SSIMs of the motion-corrected images, and we found that Pix2Pix was significantly higher than U-Net (*p* < 0.05). Qualitative evaluation revealed that motion-corrected images generated by U-Net and Pix2Pix from artifact-free images (0_none) were all point as 5. Furthermore, there was no difference in the qualitative evaluation between Pix2Pix and U-Net when the rotation angle was 1°_30 (observer 1; *p* = 0.79, observer 2; *p* = 0.53). For the 2°_20 and 3°_10 motion patterns, Pix2Pix showed a significantly higher score by both observers compared to U-Net (all *p* < 0.05). The other 1.5°_25 and 2.5°_15 motion patterns, showed a significantly different higher score by one observer (1.5_25; observer 1; *p* = 0.23, observer 2; *p* < 0.05, 2.5_15; observer 1; *p* < 0.05, observer 2; *p* = 0.14). The weighted kappa test showed strong agreement between the two radiologists (*k* = 0.62).

**Table 2:** Results of SSIM analysis and qualitative evaluation when set to trained rotation angles and k-space intervals.

			SSIM	Qualitative evaluation	
				mean ± SD (median)	
	RA	KI	(mean ± SD)	Observer 1	Observer 2
artifact image vs. original image	0	None	1.00	5.00 ± 0.00 (5)	5.00 ± 0.00 (5)
	1°	30	0.37 ± 0.021	1.73 ± 0.46 (2)	2.09 ± 0.29 (2)
	2°	20	0.29 ± 0.018	1.45 ± 0.51 (1)	1.36 ± 0.49 (1)
	3°	10	0.22 ± 0.015	1.00 ± 0.00 (1)	1.00 ± 0.00 (1)
	1.5°	25	0.33 ± 0.018	1.73 ± 0.46 (2)	1.73 ± 0.46 (2)
	2.5°	15	0.25 ± 0.018	1.14 ± 0.35 (1)	1.09 ± 0.29 (1)
U-net motion-corrected image vs. original image	0	None	0.97 ± 0.0034	5.00 ± 0.00 (5)	5.00 ± 0.00 (5)
	1°	30	0.93 ± 0.012	4.59 ± 0.50 (5)	4.82 ± 0.39 (5)
	2°	20	0.91 ± 0.011	3.45 ± 0.51 (3)	3.91 ± 0.29 (4)
	3°	10	0.88 ± 0.016	2.95 ± 0.21 (3)	3.18 ± 0.39 (3)
	1.5°	25	0.91 ± 0.0095	3.82 ± 0.39 (4)	3.77 ± 0.43 (4)
	2.5°	15	0.88 ± 0.014	3.00 ± 0.31 (3)	3.36 ± 0.49 (3)
Pix2Pix motion-corrected image vs. original image	0	None	0.98 ± 0.0035	5.00 ± 0.00 (5)	5.00 ± 0.00 (5)
	1°	30	0.94 ± 0.010	4.64 ± 0.49 (5)	4.73 ± 0.46 (5)
	2°	20	0.92 ± 0.011	3.95 ± 0.21 (4)	4.45 ± 0.51 (4)
	3°	10	0.89 ± 0.015	3.36 ± 0.49 (3)	3.45 ± 0.51 (3)
	1.5°	25	0.92 ± 0.011	3.95 ± 0.21 (4)	4.32 ± 0.48 (4)
	2.5°	15	0.89 ± 0.013	3.32 ± 0.48 (3)	3.59 ± 0.50 (4)

Structured similarity indexing method (SSIM), Rotation angle (RA); K-space interval (KI); Standard deviation (SD).

**Fig 8:**
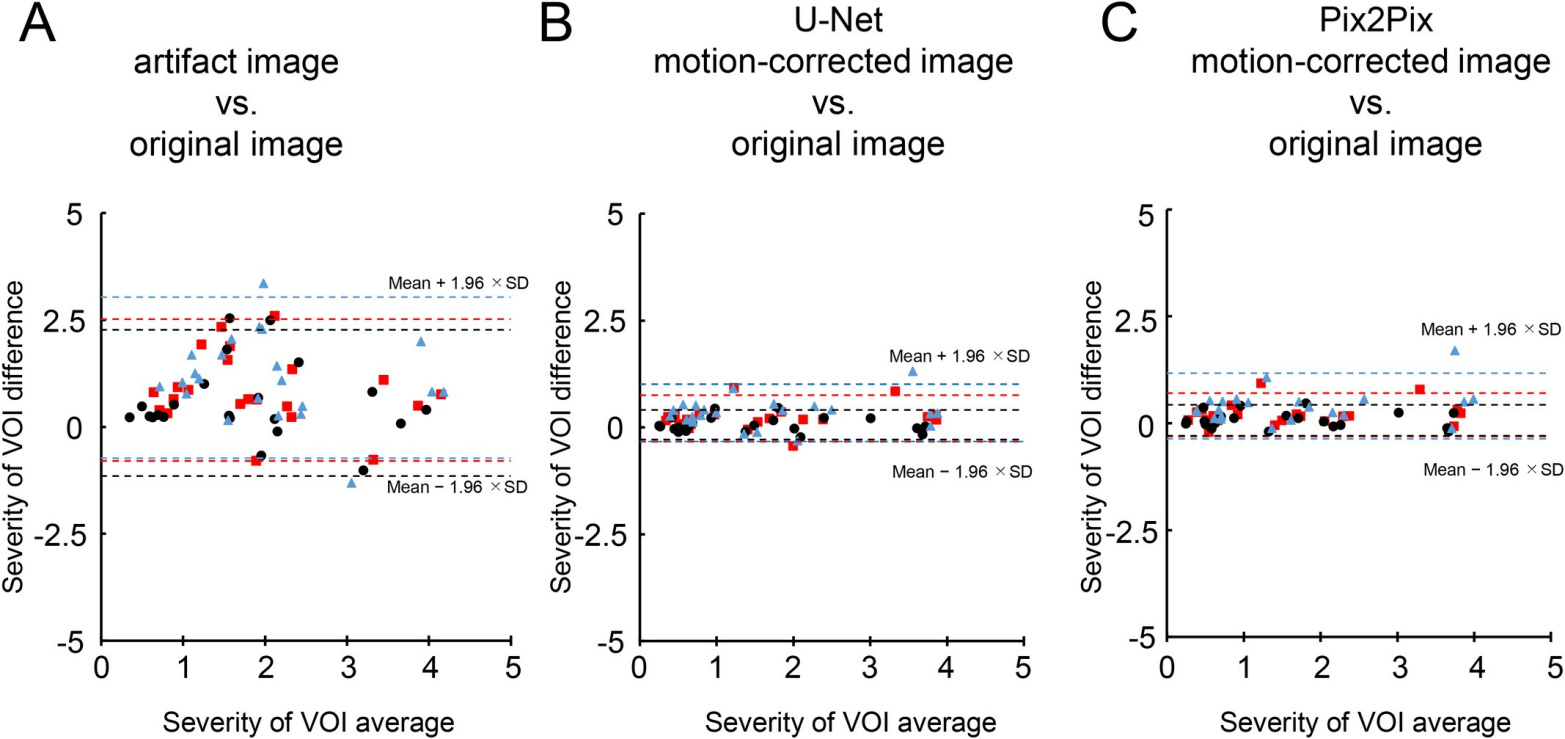
The Bland-Altman plot for VSRAD analysis results. Bland-Altman analysis results are shown for (A): severity of voxel of interest (VOI) atrophy of artifact *vs*. the original image, (B): severity of voxel of interest (VOI) atrophy of U-Net motion-corrected *vs*. the original image, (C): severity of voxel of interest (VOI) atrophy of Pix2Pix motion-corrected *vs*. the original image. The dotted line and white boxes plot show the relationship between the ±1.96 standard deviation(SD) limit of agreement of the artifact image and the original image, and the dashed line show the relationship between the ±1.96 SD limit of agreement of the motion-corrected image for U-Net or Pix2Pix and the original image. The rotation angle and k-space interval are plotted as black circles for 1°_30 pixel, red squares for 2°_20 pixel, and blue triangles for 3°_10 pixel.

The results of the Bland-Altman analysis for VSRAD of the artifact image, U-Net motion-corrected images *vs*. original images, and the Pix2Pix motion-corrected image against the original image are shown in Tables 3 and 4 and Fig 8. Artifact images demonstrated a fixed positive bias in VSRAD, which increased in accordance with the severity of the artifact. By contrast, the VSRAD results of the motion-corrected image revealed a more negligible bias than the artifact image created at the same rotation angle and k-space intervals. For example, the mean bias of the severity of VOI atrophy of the motion-corrected image 1°_30 was less than 0.1, whereas the mean bias of the motion-corrected image was almost zero when the input image was the original image (0°_none). Also, results of the VSRAD analysis using non-trained rotation angles and k-space intervals showed the same trend as the trained results (Table 4).

**Table 3:** Results of Spearman’s rank correlation coefficient and Bland-Altman analysis when set to trained rotation angles and k-space intervals.

		Motion level		Bland-Altman analysis		Spearman’s rank correlation coefficient	
		RA	KI	mean bias	Limits of agreement	*ρ*	*p*-value
artifact image vs. original image	Severity of VOI atrophy	1°	30	0.56	-1.15 ― 2.27	0.70	< 0.05
		2°	20	0.86	-0.8 ― 2.52	0.63	< 0.05
		3°	10	1.15	-0.73 ― 3.04	0.53	< 0.05
	Extent of GM atrophy	1°	30	6.27	-8.11 ― 20.65	0.35	0.072
		2°	20	7.37	0.27 ― 14.47	0.26	0.18
		3°	10	8.65	-0.2 ― 17.51	0.036	0.84
	Extent of VOI atrophy	1°	30	12.04	-32.6 ― 56.67	0.76	< 0.05
		2°	20	20.41	-23.35 ― 64.17	0.75	< 0.05
		3°	10	24.72	-23.37 ― 72.81	0.64	< 0.05
U-net motion-corrected image vs. original image	Severity of VOI atrophy	0°	none	0.031	-0.092 ― 0.155	1.00	< 0.05
		1°	30	0.061	-0.29 ― 0.41	0.98	< 0.05
		2°	20	0.21	-0.33 ― 0.76	0.97	< 0.05
		3°	10	0.34	-0.33 ― 1.01	0.94	< 0.05
	Extent of GM atrophy	0°	none	0.23	-0.18 ― 0.64	1.00	< 0.05
		1°	30	0.9	-0.38 ― 2.18	0.96	< 0.05
		2°	20	2.21	0.32 ― 4.09	0.94	< 0.05
		3°	10	2.83	0.38 ― 5.28	0.91	< 0.05
	Extent of VOI atrophy	0°	none	1.13	-2.65 ― 4.91	0.99	< 0.05
		1°	30	0.47	-9.79 ― 10.73	0.98	< 0.05
		2°	20	3.8	-13.27 ― 20.88	0.96	< 0.05
		3°	10	5.31	-12.78 ― 23.41	0.95	< 0.05
Pix2Pix motion-corrected image vs. original image	Severity of VOI atrophy	0°	none	0.027	-0.084 ― 0.14	0.99	< 0.05
		1°	30	0.067	-0.3 ― 0.43	0.97	< 0.05
		2°	20	0.2	-0.31 ― 0.7	0.95	< 0.05
		3°	10	0.4	-0.37 ― 1.17	0.94	< 0.05
	Extent of GM atrophy	0°	none	0.006	-0.391 ― 0.4	0.98	< 0.05
		1°	30	0.87	-0.37 ― 2.1	0.97	< 0.05
		2°	20	2.22	0.5 ― 3.95	0.94	< 0.05
		3°	10	3.18	1.16 ― 5.19	0.92	< 0.05
	Extent of VOI atrophy	0°	none	0.7	-2.42 ― 3.83	1.00	< 0.05
		1°	30	1.4	-6.58 ― 9.38	0.99	< 0.05
		2°	20	4.52	-9.86 ― 18.9	0.94	< 0.05
		3°	10	7.3	-10.96 ― 25.55	0.95	< 0.05

Rotation angle (RA); K-space interval (KI); Voxel of interest (VOI); Gray matter (GM). Statistical processing was performed using Spearman’s rank correlation coefficient with a significance level of 0.05.

**Table 4:** Results of Spearman’s rank correlation coefficient analysis and Bland-Altman analysis when set to non-trained rotation angles and k-space intervals.

				Bland-Altman analysis		Spearman’s rank correlation coefficient	
		RA	KI	mean bias	limits of agreement	*ρ*	*p*-value
artifact image vs. original image	Severity of VOI atrophy	1.5°	25	0.88	-2.1 ― 3.86	0.51	< 0.05
		2.5°	15	1.33	-2.4 ― 5.06	-0.094	0.68
	Extent of GM atrophy	1.5°	25	7.05	-2.67 ― 16.78	0.35	0.11
		2.5°	15	11.35	-4.4 ― 27.1	-0.32	0.14
	Extent of VOI atrophy	1.5°	25	19.24	-46.32 ― 84.8	0.58	< 0.05
		2.5°	15	18.35	-67.62 ― 104.31	-0.032	0.89
U-net motion-corrected image vs. original image	Severity of VOI atrophy	1.5°	25	0.25	-0.16 ― 0.65	0.98	< 0.05
		2.5°	15	0.47	-0.57 ― 1.5	0.93	< 0.05
	Extent of GM atrophy	1.5°	25	2.55	0.69 ― 4.41	0.95	< 0.05
		2.5°	15	3.21	0.19 ― 6.24	0.85	< 0.05
	Extent of VOI atrophy	1.5°	25	4.49	-7.57 ― 16.54	0.96	< 0.05
		2.5°	15	11.9	-21.4 ― 45.19	0.91	< 0.05
Pix2Pix motion-corrected image vs. original image	Severity of VOI atrophy	1.5°	25	0.22	-0.27 ― 0.71	0.97	< 0.05
		2.5°	15	0.56	-0.57 ― 1.69	0.87	< 0.05
	Extent of GM atrophy	1.5°	25	2.26	0.71 ― 3.8	0.97	< 0.05
		2.5°	15	3.47	0.89 ― 6.05	0.88	< 0.05
	Extent of VOI atrophy	1.5°	25	3.71	-8.64 ― 16.06	0.97	< 0.05
		2.5°	15	13.46	-22.87 ― 49.8	0.9	< 0.05

Rotation angle (RA); K-space interval (KI); Voxel of interest (VOI); Gray matter (GM). Statistical processing was performed using Spearman’s rank correlation coefficient with a significance level of 0.05.

**Fig 9:**
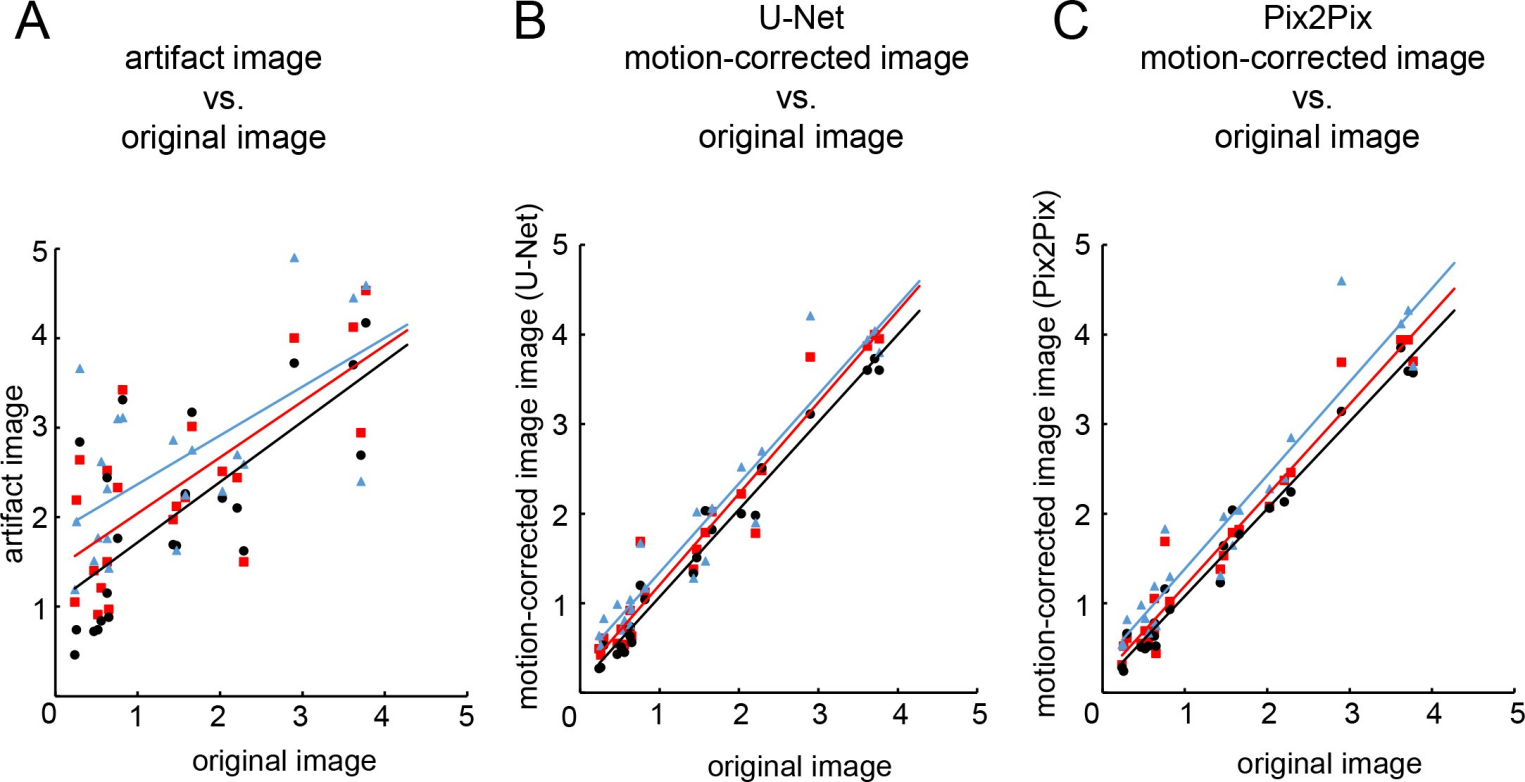
The Spearman’s rank correlation coefficient for the results of the VSRAD analysis. The results of the Spearman’s rank correlation coefficient are shown for (A): severity of voxel of interest (VOI) atrophy of artifact *vs*. the original image, (B): severity of voxel of interest (VOI) atrophy of U-Net motion-corrected *vs*. the original image and, (C): severity of voxel of interest (VOI) atrophy of Pix2Pix motion-corrected *vs*. the original image. The dotted line and white points plot show the relationship between the limits of agreement of the artifact image and the original image, and the solid line show the relationship between the limits of agreement of the motion-corrected image for U-Net or Pix2Pix and the original image. The rotation angle and k-space interval are plotted with 1°_30 pixel in black, 2°_20 pixel in red, and 3°_10 pixel in blue.

The results of the VSRAD analysis of the artifact image showed that correlations with the original image ranged from no to strong (severity of VOI atrophy: 0.53–0.70 (*p* < 0.05), extent of GM atrophy: 0.036–0.35 (*p* = 0.072–0.84 >0.05), extent of VOI atrophy: 0.64–0.76 (*p* < 0.05), all Spearman’s rank correlation coefficient). In contrast, the results of the U-Net and Pix2Pix motion-corrected image showed an almost perfect match with the original image with respect to the trained artifacts (severity of VOI atrophy, U-Net:0.94–1.00 (*p* < 0.05), Pix2Pix: 0.94–0.99 (*p* < 0.05), extent of GM atrophy, U-Net:0.91–1.00 (*p* < 0.05), Pix2Pix: 0.92–0.98 (*p* < 0.05), extent of VOI atrophy, U-Net:0.95–0.99 (*p* < 0.05), Pix2Pix: 0.94–1.00 (*p* < 0.05)) (Table 3, Fig 9), and also for the untrained artifacts (severity of VOI atrophy, U-Net:0.93–0.98 (*p* < 0.05), Pix2Pix: 0.87–0.97 (*p* < 0.05), extent of GM atrophy, U-Net:0.85–0.95 (*p* < 0.05), Pix2Pix: 0.88–0.97 (*p* < 0.05), extent of VOI atrophy, U-Net:0.91–0.96 (*p* < 0.05), Pix2Pix: 0.90–0.97 (*p* < 0.05)) (Table 4).

## Discussion

In the present study, the Pix2Pix network was trained by simulated artifact images as the input data and original images as the reference data to create motion-suppressed images (motion-corrected images) from artifact images. The results of the VSRAD analysis of the original image, the artifact image, and the motion-corrected image, were then compared. Bland-Altman analysis and Spearman’s rank correlation coefficient analysis revealed that the results of the VSRAD analysis of the motion-corrected image were more strongly correlated with the original image compared to the artifact image, suggesting that successful motion correction was obtained by Pix2Pix. Also, compared with U-net-based motion-corrected images, Pix2Pix-based motion-corrected images showed a generally higher image quality both subjectively and objectively.

The artifact images used in this study simulated two patterns of motion: rotational motion in the vertical direction and the loss of signal due to anterior-posterior motion in the sagittal plane by filling the k-space with zeros. Several studies have simulated motion artifacts using a similar method to our study [11, 24]. With this method, the motion is simulated in k-space for each slice to ensure that the slice positions of the original image and the artifact image are the same. However, the slice positions would not be identical when combining images with real motion and without motion. Therefore, we considered that the combination of the original image and the simulated artifact image is the best combination for teacher image and training image in training our network. In addition, the total number of images used for the present study was 47872 paired images for the training group. This number is sufficiently larger than that of a similar study using Pix2Pix for medical images, and it is considered to be sufficient number of images necessary for effective training [16, 25].

In the present study, we compared motion correction between GAN and CNN using U-Net, which was used to generate Pix2Pix. U-Net learns the differences between input and output images as a loss function. Therefore, U-Net learns the relationship between two images by referring to the whole image. In contrast, Pix2Pix uses PatchGAN as a discriminator, learning the true or false pair relationship of the input image by referring to the patch image, and learning a generator whose discrimination accuracy is a loss function. For these reasons, the learning methods are different even though the same network structure is used to generate the images.

Mode collapse is one of the reasons that GANs are generally considered to have poor learning stability [26]. Only one of the generators or discriminators learns quickly, which will terminate the learning progress of the other network and prevent the GAN from learning properly. In contrast, we trained our network by updating the generator and discriminator in turns. Quantitative evaluation using SSIM showed that Pix2Pix was significantly higher than U-Net, and Pix2Pix enabled improved motion correction compared to U-Net. In addition, the qualitative evaluation showed that Pix2Pix scored significantly higher than U-Net for the 2°_20 and 3°_10 motion patterns for both observers. For the 1.5_25 and 2.5_15 movements, one observer-rated Pix2Pix equal (no significant difference) to U-Net and the other observer significantly higher. Evaluating each patch image improves the learning accuracy for high-frequency components [15]. These results suggest that the training of the generators and discriminators is well-balanced and that Pix2Pix could learn motion correction with high accuracy and high-frequency components of the images, which correspond to the fine structure of images.

Large positive fixation biases were observed in the artifact images in the present study. A study of automated gray matter volume measurement reported that more significant motion artifacts resulted in smaller gray matter volumes [7]. This suggests that the VSRAD analysis may point to large GM atrophy in cases of motion artifacts and may overestimate the degree of atrophy. The Bland-Altman analysis revealed that the severity of VOI atrophy, the extent of GM atrophy, and the extent of VOI atrophy had a mean bias close to zero in the limits of agreement. This suggests that the motion-corrected images created by Pix2Pix could reduce the effects of motion artifacts and overestimation of atrophy. However, with large rotations and small k-space spacing, the VSRAD analysis results far from zero. Therefore, Pix2Pix can improve the analysis results for artifact images with large motion, yet it cannot completely remove the motion effect.

The results of the Spearman’s rank correlation coefficient analysis between the original *vs*. artifact images showed a lower correlation for the extent of GM atrophy compared to the severity of VOI atrophy and the extent of VOI atrophy. The severity and extent of VOI atrophy in the VSRAD analysis set the medial temporal regions (hippocampus, amygdala, and entorhinal cortex) as the regions of interest, while the extent of GM atrophy measured the gray matter of the whole brain. In the present study, the k-space was rotated from the center coordinate to create an artifact image. The severity and extent of VOI atrophy measures the medial temporal region close to the central coordinate, i.e., the region with the small movement. In contrast, the extent of GM atrophy measures the gray matter of the whole brain, including the gray matter with larger movement, resulting in a lower correlation with the original image. By contrast, the Spearman’s rank correlation coefficient between the original and the motion-corrected images by Pix2Pix were all very high: 0.87–0.99 for the severity of VOI atrophy, 0.88–0.98 for the extent of GM atrophy, and 0.90–1.00 for the extent of VOI atrophy, regardless of the rotation angle and k-space interval. This suggests that the present Pix2Pix network could learn to reduce artifacts stemming from both large and small movements.

We believe that the Pix2Pix network offers significant advantages in correcting motion artifacts in the VSRAD analysis. Firstly, the mean±SD of the severity of VOI atrophy in the healthy elderly group was reported to be 0.94±0.32 [27]. In contrast, severity of VOI atrophy was found to be significantly different in the mild cognitive impairment and AD groups, 1.16±0.79 and 1.88±1.07, respectively [28]. Our results showed that the mean bias of the severity of VOI atrophy was 0.067 even for images with weak artifacts (1_30). Therefore, it would be challenging to differentiate mild AD cases from patients with normal to mild cognitive impairment should the input image of VSRAD analysis contain artifacts. However, the mean bias of the severity of VOI atrophy of the motion-corrected image (1_30) was minimal (severity of VOI atrophy < 0.1), which may allow classification in such cases. Secondly, when the original image was used as the input image (0°_none), the average bias was nearly zero (severity of VOI atrophy: 0.027), suggesting negligible effects upon VSRAD analysis. Consequently, this means that both images with and without motion could be used as the input images for the Pix2Pix network. Finally, even for non-trained rotation angles and k-space intervals, Spearman’s rank correlation coefficient showed a significant correlation of more than 0.87. This shows that our trained network could achieve sufficient motion correction even in cases where the pattern of motion artifacts does not perfectly match the training data. Additionally, the advantages of using Pix2Pix in medical practice are as follows. First, when satisfactory MR images cannot be obtained from patients due to involuntary body movements, VSRAD analysis might be efficiently performed. Second, the risk of overdiagnosis can be reduced, thus reducing unnecessary additional testing and unnecessary treatment modalities. Finally, because Pix2Pix can be used for historical images, it might be possible to measure changes in AD atrophy over time that could not be analyzed in the VSRAD analysis due to motion artifacts.

Our study has several limitations. First, we used supervised learning in CNN and GAN training, a set of motion artifact images, and motion-less images. However, it was very difficult to acquire two types of images—one with motion and the other motion-less—from all AD patients. Therefore, the created artifact images were not real motion artifact images, so it remains unclear whether our findings results could be translated to clinical practice. Secondly, this study simulated vertical rotational and anterior-posterior-motion movements in the sagittal plane but did not examine left-right motion in the coronal plane. Thirdly, we did not evaluate the relationship between VSRAD score and severity classification of AD. Fourth, k-space under-sampling can reduce motion artifacts by minimizing the acquisition time. However, we did not acquire such images in this retrospective study, thus comparison between the image quality of under-sampled images and Pix2Pix motion-corrected images was not possible. Finally, the present study was performed in a single institution and the MR image data were obtained by a single machine. In the future, we would like to investigate the usefulness of similar motion correction techniques for data stored at other facilities and on other image databases.

## Conclusion

In conclusion, the VSRAD analysis tended to overestimate atrophy in images with artifacts. After reconstructing the artifact images using the Pix2Pix network to reduce motion artifact, the overestimation of atrophy was improved dramatically. We suggest that motion correction using Pix2Pix can be useful for VSRAD analysis.
